# Pre-morbid Commonalities Among Patients Undergoing Emergent Cricothyrotomy at a Tertiary Care Level 1 Trauma Center: Categorizing Our Experience

**DOI:** 10.7759/cureus.92157

**Published:** 2025-09-12

**Authors:** Reshma Modi, Yekaterina Shapiro, Shivani Raizada, Kathy Zhang, Bailey Balouch, Donald Solomon

**Affiliations:** 1 Otolaryngology-Head and Neck Surgery, Cooper Medical School of Rowan University, Camden, USA; 2 Otolaryngology-Head and Neck Surgery, Cooper University Hospital, Camden, USA

**Keywords:** airway obstruction, cricothyrotomy, difficult airway, emergency airway management, head and neck cancer, intubation failure

## Abstract

Introduction: Emergent cricothyrotomy is a lifesaving surgical procedure employed when conventional intubation fails. Scenarios requiring cricothyrotomy are uncommon, and many trainees have limited experience with the procedure. As “cannot intubate, cannot oxygenate” (CICO) scenarios demand rapid intervention, it is crucial that providers are able to determine which patients are at risk for failed intubation and, when necessary, quickly proceed to a surgical airway. This study investigates pre-morbid commonalities among patients who underwent cricothyrotomy at a tertiary care level 1 trauma center to improve the identification of difficult intubation scenarios that may require cricothyrotomy.

Methods: A retrospective analysis of the demographic, clinical, and procedural data of 32 patients who underwent emergent cricothyrotomy at our institution between 2014 and 2025 was performed. Descriptive statistics were analyzed to characterize the patient cohort.

Results: Common pre-morbid conditions in our series included chronic obstructive pulmonary disease (COPD), asthma, and head and neck cancer. The leading indications for attempted intubation were angioedema, obstructive tumor, trauma, and acute respiratory failure. Laryngoscopy revealed airway edema and blood as the primary factors obscuring the airway, preventing intubation and prompting the need for cricothyrotomy.

Conclusion: Patients with limited respiratory reserve capacity, such as in COPD and asthma, as well as those with distorted airway anatomy secondary to malignancy, radiation fibrosis, or post-surgical changes, are at higher risk for failed intubation attempts leading to eventual cricothyrotomy. The early recognition of risk factors may lead to improved airway management planning, which, in some cases, may mitigate the need for emergent cricothyrotomy.

## Introduction

Emergent cricothyrotomy is a potentially lifesaving surgical procedure performed in the setting of a “cannot intubate, cannot oxygenate” (CICO) scenario. The creation of a direct incisional airway through the cricothyroid membrane, which is anatomically bordered superiorly by thyroid cartilage and inferiorly by the cricoid cartilage, enables the rapid re-establishment of airway access for ventilation [1]. This technique is considered the final step in the difficult airway algorithm, after conventional intubation and supraglottic ventilation techniques have failed [2]. Cricothyrotomy is preferred over tracheostomy in emergent settings, due to the ability to quickly cannulate the airway through the cricothyroid membrane while avoiding the highly vascular thyroid gland [1].

Failure to intubate may be encountered in the setting of facial trauma, massive oropharyngeal hemorrhage, or airway obstruction caused by angioedema, anaphylaxis, burns, neoplastic disease, foreign bodies, or significant laryngotracheal stenosis [1,3]. Patients with complex otolaryngologic histories, including head and neck malignancy, prior radiation therapy, surgical neck dissection, or tumor resection, may be particularly predisposed to failed intubation due to altered anatomy and reduced tissue compliance. In addition, those patients with reduced respiratory reserve capacity, such as in the face of chronic obstructive pulmonary disease (COPD) or asthma, may not be able to tolerate multiple failed attempts at intubation, hence necessitating cricothyrotomy.

The incidence of cricothyrotomy during urgent or emergent intubation is low, ranging from 0.23% in hospital settings to 0.7% in prehospital trauma care [3,4]. Due to the low incidence of CICO situations, physicians might have limited experience with obtaining a surgical airway, which can delay the decision to proceed with cricothyrotomy or result in further complications. One survey of graduating emergency medicine residents indicated that while 22% had performed a cricothyrotomy on a living patient, the vast majority had experience only with recently deceased patients, simulators, or animal models [5]. Success rates of both intubation and cricothyrotomy may also be influenced by provider specialty and experience level [4,6]. Consequently, the awareness of the characteristics and comorbidities that might predispose a patient to unsuccessful intubation may improve physician preparedness and surgical outcomes for those for whom early cricothyrotomy is a lifesaving measure.

The objective of this retrospective study is to identify pre-morbid conditions and presenting factors that predispose patients to failed intubation and emergent cricothyrotomy at a tertiary care level 1 trauma center. Specifically, we sought to characterize the demographic and clinical profiles of patients requiring emergent cricothyrotomy, identify common pre-morbid conditions and airway findings that contribute to failed intubation, and evaluate the clinical settings, providers, and techniques associated with cricothyrotomy outcomes. We aim to use this data to better predict patient risk of CICO scenarios and optimize airway management strategies.

This article was previously posted to the Authorea preprint server on May 13, 2025.

## Materials and methods

This study was reviewed and approved by the Cooper Institutional Review Board (approval number: 23-141). The electronic health record was queried to identify all emergent cricothyrotomy procedures performed between 2014 and 2025. Adult patients who underwent cricothyrotomy before transfer or during admission at our institution were included. Subjects were excluded if they were below the age of 18 years. Cases were identified retrospectively through chart review and subsequently coded for analysis.

The following data was collected through chart review: patient demographics, including age, sex, body mass index (BMI), and medical comorbidities, such as COPD and asthma, and previous otolaryngologic history (e.g., head and neck cancer and subsite, radiation treatment, prior head and neck surgery, and a history of prior tracheostomy). The mechanism of injury, Glasgow Coma Scale (GCS) on presentation, and reason for cricothyrotomy were collected. Circumstances surrounding attempted intubation were also collected, including which providers were involved, the location of attempted intubation (e.g., emergency department {ED}, general medical/surgical floor, and intensive care unit {ICU}), and the reason for inability to intubate. Details of cricothyrotomy were noted, including providers involved and the use of surgical or percutaneous technique. For the purpose of this study, surgical cricothyrotomies were defined as the use of a scalpel to incise skin and cricothyroid membrane, whereas percutaneous cricothyrotomies involved the use of a needle or dilator to create a smaller passage.

SPSS version 29.0 (IBM Corp., Armonk, NY) was used to calculate descriptive statistics.

## Results

A total of 32 patients who underwent cricothyrotomy were included in this retrospective review. The average age was 56.8 ± 15.3 years (range: 16-87). Twenty-one (65.6%) patients were men, and 11 (34.4%) were women. The average body mass index (BMI) was 28.9 ± 10.0 kg/m^2^ (range: 16.0-55.4). COPD (43.8%) and asthma (25.0%) were the most common comorbidities in our study population.

There were nine patients (28.1%) with a history of head and neck cancer, and 33.3% of patients with head and neck cancer had not yet undergone treatment. Four patients with oncologic history (44.4%) had primary carcinoma of the oral cavity, two of which also had extensive obstructive involvement of the oropharynx, laryngopharynx, and/or nasopharynx. Two patients had a history of primary oropharyngeal cancer, two patients had a history of laryngeal cancer, and one patient had a history of nasopharyngeal cancer. Patients in this cohort also had a history of radiation to the neck (18.8%), head and neck surgery (12.5%), and prior tracheostomy (9.4%) (Table 1).

**Table 1:** Patient demographics and pertinent medical history COPD: chronic obstructive pulmonary disease

Demographics	
Patient Age (Years) (Mean ± SD)	56.8 ± 15.3
Male Gender (n, %)	21, 65.6%
Body Mass Index (Mean ± SD)	28.9 ± 10.0
Current Tobacco Use (n, %)	9, 28.1%
Former Tobacco Use (n, %)	13, 40.6%
Pack Years (Mean ± SD)	26.0 ± 25.1
Alcohol Use (n, %)	5, 15.6%
Other Substance Use (n, %)	3, 9.4%
Pertinent Past Medical History	
COPD (n, %)	14, 43.8%
Asthma (n, %)	8, 25.0%
Head and Neck Cancer (n, %)	9, 28.1%
Radiation to the Neck (n, %)	6, 18.8%
Neck Surgery (n, %)	4, 12.5%
Prior Tracheostomy (n, %)	3, 9.4%
Cervical Spine Surgery (n, %)	1, 3.1%

The most common presenting findings that led to attempted intubation were angioedema (18.8%), head and neck cancer with obstructive tumor arising from the upper aerodigestive tract (18.8%), trauma (12.5%, specifically facial trauma in 6.3%), and acute respiratory failure (12.5%) (Table 2). Glasgow Coma Scale (GCS) was on average 9.0 ± 5.2 (range: 3-15) at presentation.

**Table 2:** Presenting problem leading to the need for intubation

Presenting Problem	n, %
Angioedema	6, 18.8%
Obstructive Aerodigestive Tract Tumor	6, 18.8%
Trauma	4, 12.5%
Acute Respiratory Failure	4, 12.5%
Diabetic Ketoacidosis	2, 6.3%
Cardiac Arrest	2, 6.3%
Other Upper Airway Obstruction	2, 6.3%
Encephalopathy	1, 3.1%
Neck Hematoma	1, 3.1%
Bleeding Esophageal Varices	1, 3.1%
Ludwig’s Angina	1, 3.1%
Status Epilepticus	1, 3.1%
Planned Intubation for General Anesthesia	1, 3.1%

The hospital settings where attempted intubation and subsequent cricothyrotomy took place included the emergency department (ED) (40.6%), hospital floors (25.0%), intensive care unit (ICU) (15.6%), and the operating room (6.3%). In 9.4% of cases, the setting was not recorded. Intubation was attempted in all cases. Providers who attempted intubation included anesthesia attendings (37.5%), ED attendings (31.3%), critical care attendings (25.0%), ED residents (18.8%), critical care fellows (12.5%), anesthesia residents (6.3%), trauma surgery attendings (3.1%), certified registered nurse anesthetists (CRNA) (3.1%), and emergency medical technicians (EMT) (3.1%). Unsuccessful intubation was attributed to a variety of factors, including airway edema (59.4%), blood in the airway obstructing visualization (21.9%), laryngeal injury (15.6%), trismus (15.6%), distorted anatomy (9.4%), anterior-positioned airway (3.1%), and massive emesis during intubation (3.1%) (Table 3).

**Table 3:** Clinical and laryngoscopic findings

Clinical and Laryngoscopic Findings	n, %
Airway Edema	19, 59.4%
Blood in the Airway	7, 21.9%
Laryngeal Injury	5, 15.6%
Trismus	5, 15.6%
Distorted Anatomy	3, 9.4%
Anterior Airway	1, 3.1%
Massive Emesis	1, 3.1%

Cricothyrotomy was performed for all patients included in this series. The providers who performed the cricothyrotomy included trauma surgery attendings (28.1%), ED attendings (18.8%), ED residents (9.4%), surgical residents (6.3%), critical care attendings (6.3%), otolaryngology attendings (6.3%), trauma fellows (3.1%), or critical care fellows (3.1%). Cricothyrotomy was performed surgically in nearly all cases but percutaneously in one case.

As assessed clinically, cricothyrotomy successfully re-established an airway in 100% of patients in this series. Seven patients (21.9%) underwent cricothyrotomy, alongside cardiopulmonary resuscitation with successful return of spontaneous circulation (ROSC). Six of these patients recovered and later underwent cricothyrotomy to tracheostomy conversion. Overall, cricothyroidotomy to tracheostomy conversion was performed in 27 (84.4%) patients in this cohort. Five patients died prior to conversion. Postoperatively, three patients (9.4%) experienced surgical site infection, and two patients were found to have tracheal or cricoid cartilage fractures (6.3%) during their admission. The in-hospital mortality rate for patients having undergone cricothyrotomy in this series was 21.9%.

## Discussion

This retrospective study characterizes the demographics, comorbidities, clinical presentation, and upper airway findings of patients who underwent emergent cricothyrotomy at a level 1 tertiary care trauma center. The majority of patients were middle-aged men, consistent with reported demographics in prior studies [3]. Prior studies have also identified higher BMI, specifically obesity (BMI > 30 kg/m^2^), as a risk factor for the need for a surgical airway due to increased airway adiposity and decreased expiratory reserve volume [7,8]. The average BMI for patients in this cohort was 28.9 kg/m^2^, which is similar to the average BMI of the general population, suggesting that other primary patient factors were more relevant [9]. Our findings highlight that while obesity is a well-recognized risk factor, institutional differences and other patient comorbidities may play a more significant role in certain populations.

Comorbid pulmonary disease was common, and two-thirds of patients were current or former smokers. Pulmonary history might increase the likelihood of intubation due to ventilation-perfusion mismatch but is unlikely to account for intubation failure. The majority of these patients also had altered airway anatomy (i.e., tumor, laryngeal injury, and radiation history), obstruction by blood or emesis, or angioedema, which more likely contributed to intubation failure. The remaining patients required cricothyrotomy due to upper airway edema of unknown etiology.

The leading presentations requiring intubation were angioedema and obstructive aerodigestive tract malignancy, followed by trauma and acute respiratory failure. Patients presented with a mean GCS of 9, indicating moderately impaired consciousness. Among experienced providers, the literature reports that first-attempt intubation of patients presenting with angioedema is successful in 82% of cases, with an overall success rate of 99% [10]. However, success varies with provider experience, technique, and available equipment (e.g., fiberoptic bronchoscopy or videolaryngoscopy) due to rapid supraglottic swelling, distorted airway anatomy, and limited visualization associated with angioedema [11,12]. The high prevalence of angioedema in this cohort may be attributable to this variability and points to the significant severity of angioedema in the patients in our series that underwent cricothyroidotomy.

The most frequent head and neck cancer subsite was the oral cavity. Large oral cavity tumors can be physically obstructive, and if medial or lateral pterygoid invasion is present, this can lead to trismus as well. Both factors may create a situation where orotracheal intubation is unsuccessful. Additionally, three patients in this cohort had glottic or supraglottic tumors causing near-total airway occlusion. In all three cases, cricothyrotomy provided temporary stabilization, allowing for sufficient ventilation until a revision tracheotomy could be performed. Of the seven patients with documented staging, five had advanced T4 cancers.

Laryngoscopic evaluation demonstrated that airway edema was by far the leading cause of intubation failure in our cohort. Impaired oral access due to trismus and visual obstruction by blood, emesis, or distorted anatomy (i.e., tumor compression, post-surgical changes, and laryngeal injury) were also implicated in the need for a surgical airway, as has been observed in other similar studies [6]. The visualization of the larynx can provide quick confirmation of whether traditional intubation is possible and, therefore, can both reduce unnecessary cricothyrotomies and minimize cricothyrotomy delay if awake tracheostomy is not a viable option due to patient decompensation.

Multiple patients in this cohort had a history of head and neck cancer, radiation treatment, or neck and cervical spine surgeries. As prior studies have indicated, altered cervical anatomy, due to malignancy, fibrosis, and post-surgical changes, is a key predictor of a difficult airway and increases the risk of failed intubation secondary to limited visualization related to trismus, limited neck mobility or extension, and decreased upper airway compliance [7].

Most cricothyrotomies in this study were performed surgically, in contrast to other studies, which reported predominantly percutaneous techniques [3]. While the emergency department was the most common setting for cricothyrotomy, a substantial number occurred on hospital floors and in the ICU, underscoring the need for surgical airway preparedness in all settings.

This study is limited by its single-center analysis and relatively small sample size, which restricts generalizability. In addition, without a control group, it is not possible to quantify the relative risk of cricothyrotomy given any one preexisting medical condition or presenting problem. Documentation in the electronic medical record also varied and, in some cases, may not have captured the full rationale for proceeding to cricothyrotomy or indicated whether residents or CRNAs attempted intubation prior to the attending physician. Furthermore, skill levels may differ among airway teams, making it uncertain whether an intubation failure in one setting would yield the same outcome under another provider team. For instance, previous literature has found that non-anesthesiologists are twice as likely to fail intubation and proceed to cricothyrotomy as anesthesiologists, suggesting that some cricothyrotomies may be performed unnecessarily [4]. However, in this series, attending anesthesiologists were the most frequent providers present at airway emergencies, which may indicate that at our institution, the decision to proceed to surgical airway is not significantly influenced by provider skill level.

Cricothyrotomy successfully re-established an airway in 100% of patients, as evaluated clinically. As emergent cricothyrotomy is an uncommon procedure, this series contributes to currently limited data in the literature regarding pre-morbid conditions and airway findings in a US trauma center population.

## Conclusions

This study demonstrates that patients with distorted airway anatomy, such as those with head and neck malignancy, radiation fibrosis, and post-surgical changes, are at heightened risk for failed intubation and may ultimately require surgical intervention. Airway edema was the most common contributor to intubation failure in our study. We did not note morbid obesity as a significant risk factor for cricothyrotomy in contrast to previously reported series. The early recognition of risk factors for “cannot oxygenate, cannot intubate” scenarios, as noted above, may improve airway management planning, which in turn could potentially reduce the need for cricothyrotomy. These findings may inform future airway triage protocols and pre-intubation risk assessment tools in trauma and ICU settings. The early identification of patients with significant airway edema, advanced malignancy, or postradiation changes could prompt the earlier consideration of surgical airway and the mobilization of experienced providers, potentially improving patient safety in high-risk scenarios. The incorporation of these variables into established frameworks such as Look, Evaluate, Mallampati, Obstruction, and Neck mobility (LEMON) or Mallampati score III or IV, Apnea, Cervical spine movement limited, mouth Opening <3 cm, Coma, Hypoxemia, and non-Anesthesiologist intubator (MACOCHA) scores or adapting a pre-intubation checklist specific to high-risk patients could provide a structured approach to risk stratification and guide earlier escalation to surgical airway when indicated.
